# Factors affecting outcome in frameless non-isocentric stereotactic radiosurgery for trigeminal neuralgia: a multicentric cohort study

**DOI:** 10.1186/s13014-020-01535-1

**Published:** 2020-05-22

**Authors:** Alfredo Conti, Gueliz Acker, Antonio Pontoriero, Juliane Hardt, Anne Kluge, Alberto Cacciola, Giuseppe Iatì, Markus Kufeld, Volker Budach, Peter Vajkoczy, Giancarlo Beltramo, Stefano Pergolizzi, Achille Bergantin, Franziska Loebel, Silvana Parisi, Carolin Senger, Pantaleo Romanelli

**Affiliations:** 1 Department of Neurosurgery, Charité - Universitätsmedizin Berlin, corporate member of Freie Universität Berlin, Humboldt-Universität zu Berlin and Berlin Institute of Health, Charitéplatz 1, 10117 Berlin, Germany; 2 Charité CyberKnife Center, Charité - Universitätsmedizin Berlin, Augustenburger Platz 1, Berlin, 13353 Germany; 3 Unit of Neurosurgery, IRCCS ISNB Istituto delle Scienze Neurologiche di Bologna; Department of Biomedical and Neuromotor Sciences, Alma Mater Studiorum University of Bologna, Bologna, Italy; 4 Berlin Institute of Health (BIH), Anna-Louisa-Karsch-Str. 2, Berlin, 10178 Germany; 5 Department of Radiation Oncology, University of Messina, Messina, Italy; 6 Institute of Biometry and Clinical Epidemiology, Charité - Universitätsmedizin Berlin AND Berlin Institute of Health , Berlin, Germany; 7 Clinical Research Unit (CRU), Berlin Institute of Health, Charitéplatz 1, 10117 Berlin, Germany; 8 Fakultät III, Dep. Information & Communication, Medical Information Management, Hochschule Hannover - University of Applied Sciences and Arts, Expo Plaza 12, 30539 Hannover, Germany; 9 Department of Radiation Oncology, Charité - Universitätsmedizin Berlin, Augustenburger Platz 1, 13353 Berlin, Germany; 10 Brain Radiosurgery, CDI Centro Diagnostico Italiano, Milan, Italy

**Keywords:** CyberKnife, Neuropathic pain, Stereotactic radiosurgery, Trigeminal neuralgia, Neuropathic pain

## Abstract

**Background:**

Stereotactic radiosurgery (SRS) is an effective treatment for trigeminal neuralgia (TN). Nevertheless, a proportion of patients will experience recurrence and treatment-related sensory disturbances. In order to evaluate the predictors of efficacy and safety of image-guided non-isocentric radiosurgery, we analyzed the impact of trigeminal nerve volume and the nerve dose/volume relationship, together with relevant clinical characteristics.

**Methods:**

Two-hundred and ninety-six procedures were performed on 262 patients at three centers. In 17 patients the TN was secondary to multiple sclerosis (MS). Trigeminal pain and sensory disturbances were classified according to the Barrow Neurological Institute (BNI) scale. Pain-free-intervals were investigated using Kaplan Meier analyses. Univariate and multivariate Cox regression analyses were performed to identify predictors.

**Results:**

The median follow-up period was 38 months, median maximal dose 72.4 Gy, median target nerve volume 25 mm^3^, and median prescription dose 60 Gy. Pain control rate (BNI I-III) at 6, 12, 24, 36, 48, and 60 months were 96.8, 90.9, 84.2, 81.4, 74.2, and 71.2%, respectively. Overall, 18% of patients developed sensory disturbances. Patients with volume ≥ 30 mm^3^ were more likely to maintain pain relief (*p* = 0.031), and low integral dose (< 1.4 mJ) tended to be associated with more pain recurrence than intermediate (1.4–2.7 mJ) or high integral dose (> 2.7 mJ; low vs. intermediate: log-rank test, χ^2^ = 5.02, *p* = 0.019; low vs. high: log-rank test, χ^2^ = 6.026, *p* = 0.014). MS, integral dose, and mean dose were the factors associated with pain recurrence, while re-irradiation and MS were predictors for sensory disturbance in the multivariate analysis.

**Conclusions:**

The dose to nerve volume ratio is predictive of pain recurrence in TN, and re-irradiation has a major impact on the development of sensory disturbances after non-isocentric SRS. Interestingly, the integral dose may differ significantly in treatments using apparently similar dose and volume constraints.

## Background

Trigeminal neuralgia (TN) is the most common cranio-facial pain syndrome. Medical therapy, usually based on carbamazepine, is the first treatment option, but effective pain control often requires doses associated with severe side effects. Surgical treatment option is considered when drug therapy deems ineffective in controlling pain and/or causes severe side effects [1]; thus, surgery is often necessary. In case of a neurovascular conflict, the first line treatment is microvascular decompression (MVD), which can result in up to 100% initial pain relief [2–5]. Nonetheless, this technique is not always applicable due to a lack of neurovascular conflict, contraindications for major surgery, or patient preference. Alternative techniques aim to modulate the trigeminal nociceptive pathways either by percutaneous lesioning of the Gasserian ganglion or by irradiation of the cisternal portion of the nerve using stereotactic radiosurgery (SRS) [1, 6, 7]. Clinical experience regarding TN treatment using SRS is based mainly on single isocenter Gamma knife radiosurgery (GKS) treatments [8]. Typically GKS results in up to 92% pain relief within 12 months; however, the pain relapse rate during follow-up is remarkable [9–17]. Alternatively, radiosurgical rhizotomy can be performed using a frameless image-guided robotic technique with the CyberKnife system (CKS) to irradiate an individually contoured segment of the trigeminal nerve by delivering non-isocentric radiation beams [18–20]. So far, reported clinical results of CKS seem to be satisfactory, with initial pain relief reported in 67.0 to 97.8% of the patients [7, 21, 22]. As with the GKS series, pain relapse is the major pitfall of the radiosurgical treatments, with pain control decreasing over time [7]. The ratio of dose to nerve volume, smaller nerve volume, and low prescribed dose have been claimed as potential predictors for treatment failure after SRS [7, 23, 24]; however, large cohort studies analyzing radiation metric factors interfering with the outcome in non-isocentric SRS for TN are yet to be reported. Thus, there is a lack of evidence for standardized therapy algorithms that include the selection of relevant radiosurgical treatment parameters such as dose to the target, target nerve volume, and prescription isodose.

The aim of our study was to evaluate predictors of efficacy and safety of image-guided robotic radiosurgery in a large multicentric patient cohort.

## Methods

### Setting and study design

The patient data were retrospectively analyzed; thus, consent forms were deemed unnecessary. Data were pooled from two Italian (centers A and B: CDI Centro Diagnostico Italiano, Milan and Department of Radiation Oncology, University of Messina, Messina) and one German (center C: CyberKnife Center, Charité - Universitätsmedizin Berlin) centers. The retrospective analysis was approved by the Ethics Committee Campus Charité Mitte (EA1/233/18) and by Comitato Etico Interaziendale Messina (80/19).

### Participants

Patients with TN, as defined by the International Headache Society (2003), who were treated using non-isocentric, image-guided robotic radiosurgery for medically resistant pain between 2010 and 2016 (center A), 2013–2018 (center B), and 2011–2018 (center C) were included in this study.

### Variables

We gathered data regarding the presence of multiple sclerosis (MS), re-irradiation, decision making for SRS, clinical outcome, and therapy-associated morbidity with a focus on sensory dysfunctions. Mean and maximal treatment dose (D_mean_, D_max_), target nerve volume, prescription dose (PD), and integral dose (ID, calculated as target nerve volume × mean dose) were the parameters investigated.

### Radiosurgery treatment

**Fig. 1:**
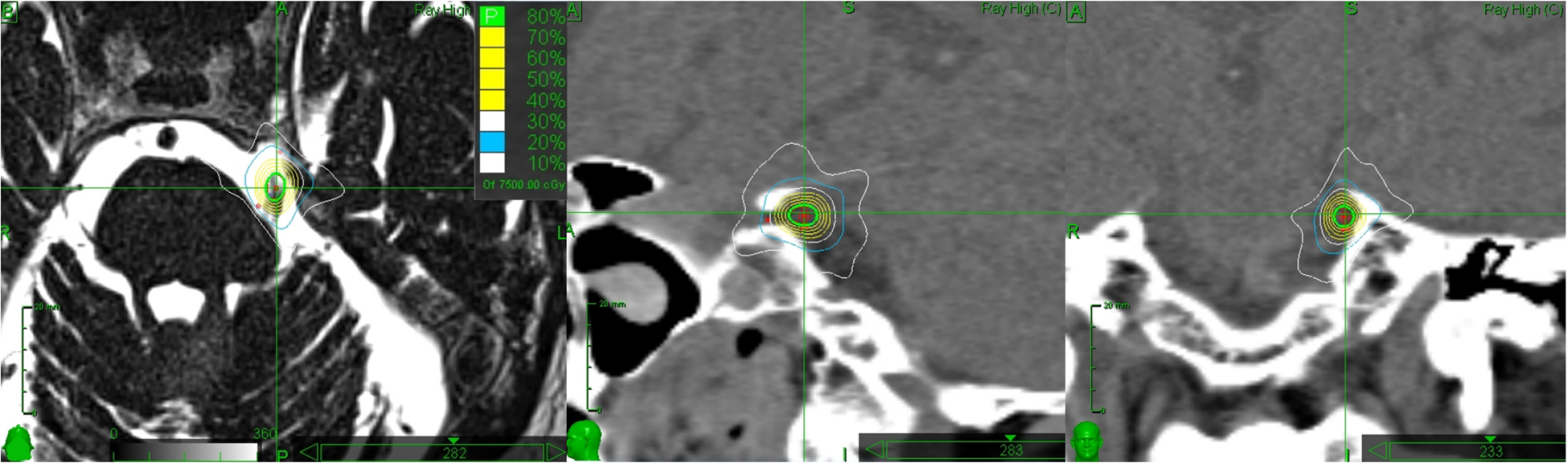
Typical CyberKnife stereotactic dose distribution for trigeminal neuralgia targeted at the cisternal portion of the nerve. left: MR CISS weighted axial image, middle and right: sagittal and coronal CT images. The dose is typically 60 Gy prescribed to the 80% isodose line

Patients were treated with SRS using a CyberKnife system (Accuray Inc., Sunnyvale, California) as described before [7]. The treatment was planned using MultiPlan (Accuray Inc.) on a native computed tomography (CT) scan (120 kV, slice thickness: ≤ 1.0 mm) and co-registered constructive interference in steady state (CISS) magnetic resonance (MR) images (T2-weighted, 3D gradient echo technique, isotropic voxel size: ≤ 1.0 × 1.0 × 1.0 mm^3^) (Fig. 1). The target nerve volume includes the complete nerve diameter over an interindividual nerve length, typically 5–6 mm of the cisternal portion of the trigeminal nerve outlined, depending on the patient’s anatomy. The 80% isodose line was then prescribed, encompassing the contoured nerve volume between the root entry zone and the area where the nerve leaves the intracranial space to enter the Meckel’s cave, while keeping a certain distance to the brainstem and temporal lobe. The treatment dose was selected according to each hospital standard. Dose constraints to organs at risk (OAR) were as follows: < 0.50 cm^3^ of the brainstem was allowed to receive 10.0 Gy with a maximum dose of 15.0 Gy in ≤0.035 cm^3^ and ≤ 0.035 cm^3^ of the temporal lobe was allowed to receive a maximum dose of 35.0 Gy. Treatment dose or volume was adjusted if the OAR constraints were not applicable due to individual trigeminal nerve anatomy or cisternal narrowness.

Treatment planning, determination of target and dose, and treatment delivery are described in detail in Additional file 1.

### Outcome assessment

Clinical follow-up was carried out 3–6 months after CK-SRS and then once yearly. The latest follow-up was included in this study. The focus of the follow-up was to verify the pain relief and new onset of facial numbness; accordingly, the endpoints analyzed were: i) pain relief, ii) occurrence of sensory disturbance, and iii) rate of pain recurrence.

### Quantitative variables

Pain and hypoesthesia evaluation were scored using the Barrow Neurological Institute (BNI) scales for pain I-V and for hypoesthesia I-IV, respectively [25]. We dichotomized the pain response as sufficient or inadequate after treatment as BNI grades I-IIIb and IV-V, respectively. Similarly, non-significant or bothersome numbness were categorized as BNI grades I-II and III-IV, respectively. Any consecutive trigeminal motor deficits were recorded.

### Statistical methods

Overall, the entire pain-free interval was examined using Kaplan Meier analysis. Pain-free intervals for 6, 12, 24, 36, 48, and 60 months were calculated. Group comparison was carried out using the log-rank test. In order to assess risk factors potentially associated with pain relapse or occurrence of numbness, univariate and multivariate Cox regression analyses were performed using clinically selected variables. The selection process of variables for the multiple regression model included two steps as described before [26].

The treatment parameters were reported as mean (standard deviation [SD]) and median. SPSS version 25.0. (Armonk, NY; IBM Corp.) was used for statistical analyses.

As this was an exploratory study where a series of group comparisons, regression analyses, and tests of normal distribution for continuous variables were performed, the two-sided *p*-values, described as significant if < 0.05, are meant as indicators and should not be interpreted as confirmatory.

## Results

### Participants

**Fig. 2:**
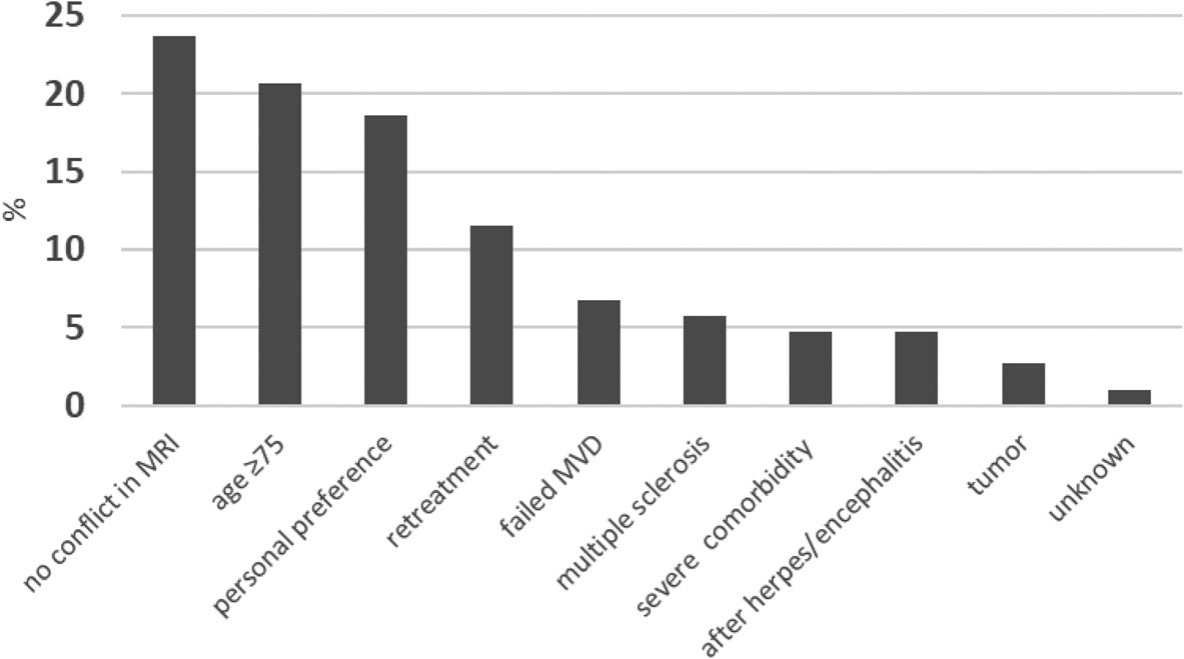
An overview of the indications for CyberKnife stereotactic radiosurgery instead of microvascular decompression (MVD)

Patient characteristics are listed in Table 1. A total of 296 TN treatments in 262 patients were included in the analysis. Center A included 144 patients (55%), center B included 79 patients (30.2%), and center C included 39 patients (14.8%). Accordingly, 34 patients (13%) needed CyberKnife-SRS re-irradiation. We analyzed the decision-making process for selecting CyberKnife-SRS instead of MVD. The most crucial reasons were missing nerve/vessel conflicts on MR images and age of the patients (age ≥ 75 years), followed by personal preference (Fig. 2).

**Table 1:** Patient demographic characteristics

	Total	Center A	Center B	Center C
Treatments (Patients)	296 (262)	176 (144)	80 (79)	40 (39)
Sex				
Male	42.9%	44.3%	42.5%	37.5%
Female	57.1%	55.7%	57.5%	62.5%
Age at treatment (years)				
Median (range)	63.8 (22.4–91.4)	60.5 (22.4–89.2)	66.9 (40.7–88.1)	66.1 (40.9–91.4)
Follow-up (months)				
Mean (SD)	40.7 (29.0)	38.4 (20.4)	58.3 (36.6)	16.4 (16.6)
Median (range)	38.0 (0.7–117.0)	38.4.0 (3.7–79.3)	49.0 (3.0–117.0)	8.0 (0.7–66.1)
Re-irradiation (n)	34	32	1	1
MS (n)	17	8	0	9

There was overall a minor female predominance (1.4:1.0). MS was diagnosed in 6.5% (*n* = 17) of the patients. The median age at therapy was 64 years (range 22–91 years).

A follow-up with at least 3 months intervals was possible in 96% of the cases. The median follow-up time to last contact was 38 months (range 0.7–117 months).

### Main results

#### Radiosurgical parameters

The treatment plans and target nerve volume, PD, D_mean_, D_max_, and ID data for the three different institutions are summarized in Table 2. The treatment parameters were overall comparable in the three centers, with differences in volume, D_max_, and ID.

**Table 2:** Summary of treatment parameters in the three centers

		n	Mean (SD)	Median	Min	Max
Volume (mm^3^)	Center A	176	29.0 (14.3)	25.0	5.0	103.3
	Center B	80	27.7 (12.3)	30.0	10.0	70.0
	Center C	40	23.8 (12.8)	20.0	10.0	60.5
	Overall	296	28.0 (13.7)	25.0	5.0	103.3
Prescription dose (Gy)	Center A	176	57.7 (6.7)	60.0	35.0	90.0
	Center B	80	57.4 (2.6)	58.0	38.0	60.0
	Center C	40	60.2 (4.1)	60.0	49.0	70.0
	Overall	296	58.0 (5.6)	60.0	35.0	90.0
Isodose (%)	Center A	176	83.0 (3.2)	83.0	73.0	90.0
	Center B	80	80.0 (0.4)	80.0	78.0	80.0
	Center C	40	80.0 (3.2)	80.0	70.0	85.0
	Overall	296	81.6 (3.1)	80.0	70.0	90.0
D_max_ (Gy)	Center A	176	70.0 (8.1)	71.3	42.7	112.5
	Center B	80	72.0 (3.3)	72.5	47.5	75.0
	Center C	40	75.7 (4.0)	75.0	70.0	87.5
	Overall	296	71.1 (7.0)	72.4	42.7	112.5
D_mean_ (Gy)	Center A	176	63.7 (7.3)	65.3	38.8	101.3
	Center B	80	64.7 (2.9)	65.3	42.8	67.5
	Center C	40	68.1 (3.8)	67.5	59.8	80.1
	Overall	296	64.5 (6.2)	65.3	38.8	101.3
Integral dose (mJ)	Center A	176	2.0 (0.9)	1.6	0.3	6.7
	Center B	80	1.8 (0.8)	1.9	0.6	4.4
	Center C	40	1.6 (0.8)	1.4	0.7	4.0
	Overall	296	1.8 (0.9)	1.6	0.3	6.7

#### Response of TN to primary CKS

**Fig. 3:**
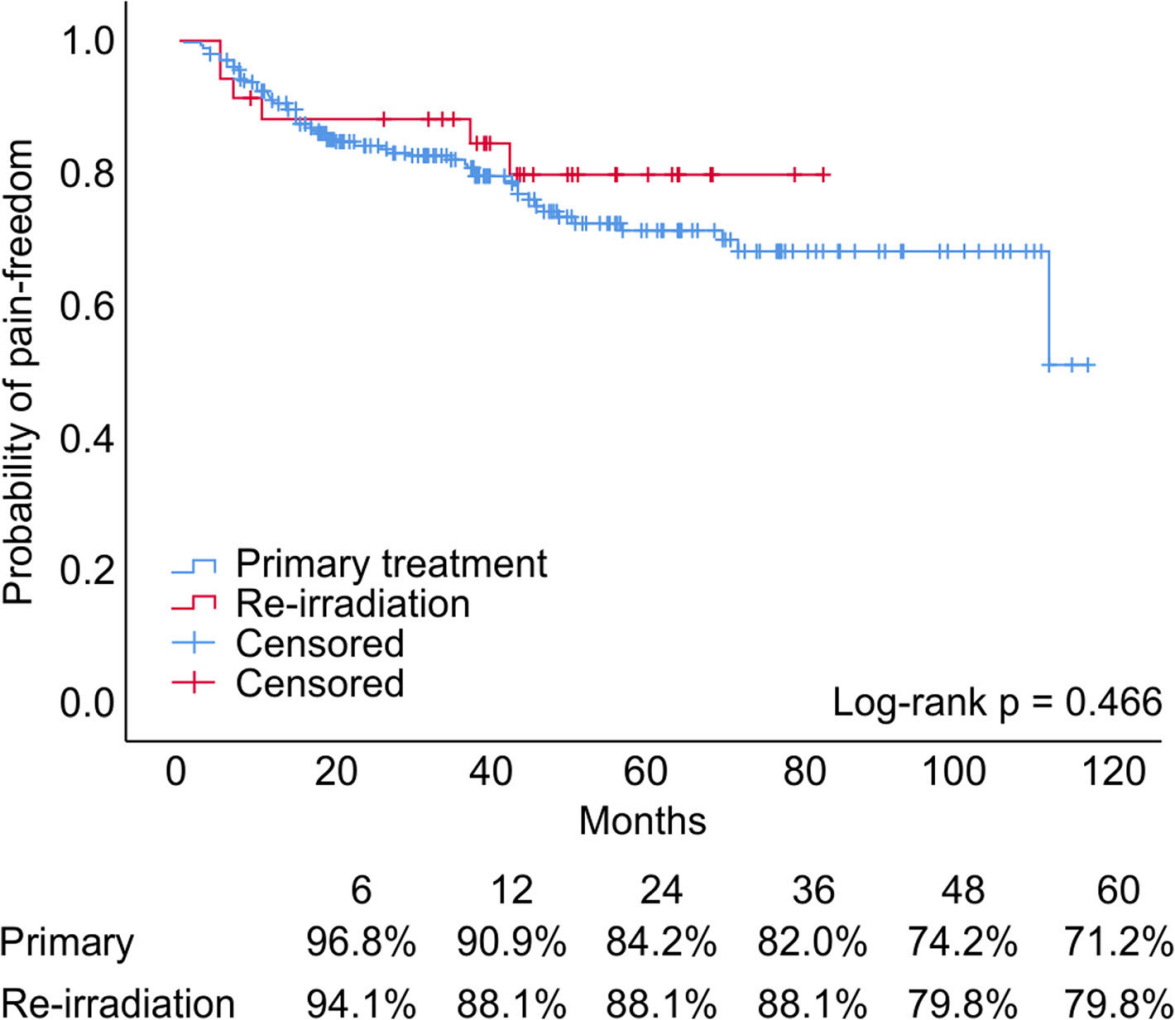
Pain relief Kaplan Meier curves for primary and re-irradiation treatments

For this analysis, we focused on the response to the first SRS treatment and excluded the retreated patients. Actuarial pain control rates could be acquired in 88.2% of the cases (231 patients). In 54.1% of the patients, full pain control (BNI I) could be achieved (Table 3). Estimated pain control rates (BNI class I-III) at 6, 12, 24, 36, 48, and 60 months were 96.8, 90.9, 84.2, 81.4, 74.2, and 71.2%, respectively (Fig. 3). Facial numbness as a side effect occurred in 18% of patients.

**Table 3:** Summary of actuarial pain control classified on the Barrow Neurological Institute (BNI) pain scale in 231 patients

BNI grade	n	%
I	125	54.1
II	25	10.8
III	33	14.3
IV	22	9.5
V	26	11.3
Total	231	100.0

Grades III a and b were combined

#### Re-irradiation

We identified 34 SRS re-irradiations in our cohort. None of the retreated patients had MS. The majority of the patients (70%) suffered from new onset numbness after re-irradiation. Actuarial pain control rates (BNI class I-III) for re-irradiation at 6, 12, 24, 36, 48, and 60 months were 91.2, 88.1, 88.1, 88.1, 79.8, and 79.8%, respectively (Fig. 3). There was, however, no statistically significant difference compared to primary therapy.

### Predictors for treatment outcome

#### Pain relief

**Fig. 4:**
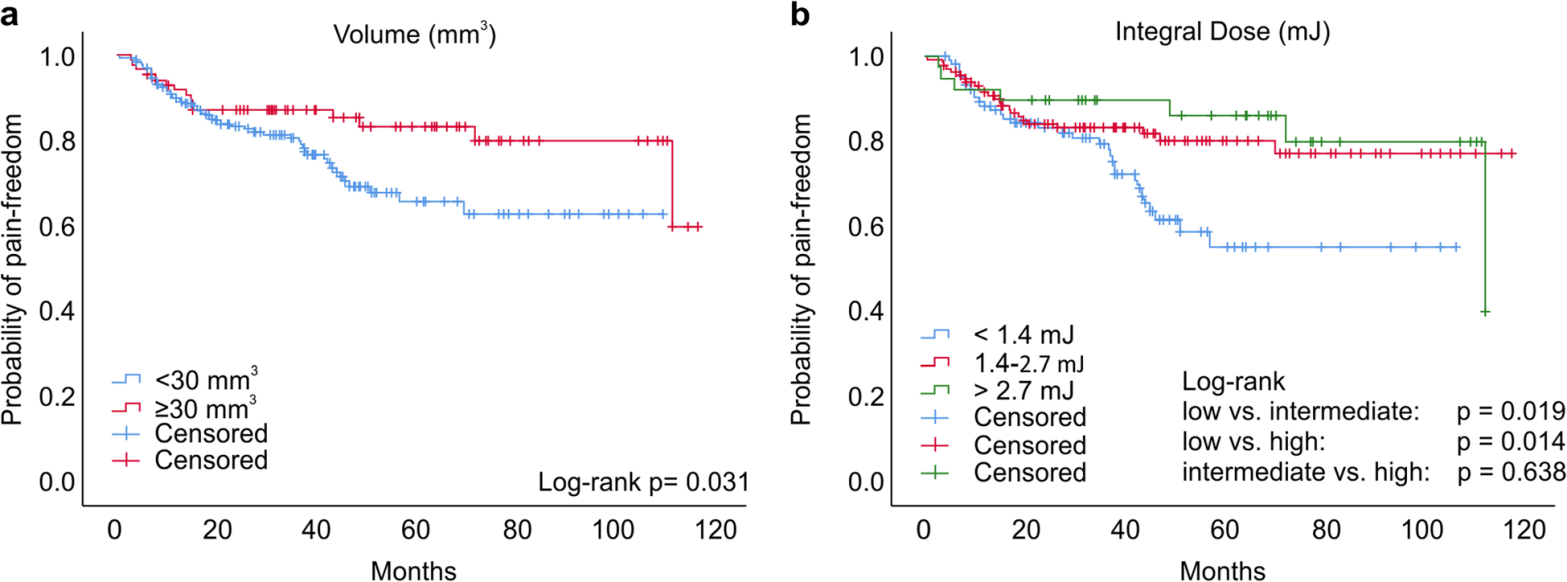
Kaplan Meier comparisons of pain-freedom probability. **a** target volume (< 30 mm^3^ vs. ≥ 30 mm^3^) and (**b**) integral dose (< 1.4 mJ; 1,4–2,7 mJ; > 2,7 mJ)

Univariate Cox regression analyses showed that lower ID, smaller nerve volume, and presence of MS were associated with treatment failure (Table 4). With regard to multicollinearity, we had to decide between D_mean_, D_max,_ ID, and volume for inclusion in the multivariate analysis. We chose mean ID based on clinical interest. In multivariate analysis, lower ID and presence of MS were the major predictors for treatment failure, while higher mean dose also emerged as a significant predictor with a slightly higher risk for pain recurrence. With regard to pain as the outcome, the multivariate Cox regression model was comparable when patients with re-irradiation and/or MS were excluded (ID: hazard ratio [HR] = 0.517, confidence interval [CI] = 0.354–0.756); D_mean_: HR = 1.038, CI = 0.999–1.079). The probability of maintaining pain relief was then compared according to the categorized treatment parameters (Table 4): volume (*<*30 vs. *≥*30 mm^3^), ID (< 1.4, 1.4–2.7, > 2.7 mJ), D_mean_ (< 63 vs. *≥* 63 Gy), D_max_ (< 72 vs. *≥* 72 Gy), and PD (< 58 vs. *≥* 58 Gy). Here, only the volume and ID comparisons resulted in significant differences. Patients with volume above 30 mm^3^ were less likely to experience pain recurrence (*<* 30 vs. *≥* 30 mm^3^; χ^2^ = 4.675, *p* = 0.031, Table 4; Fig. 4a), while patients with low ID (< 1.4 mJ) tended to suffer more and earlier pain recurrence compared to intermediate (1.4–2.7 mJ) or high ID (> 2.7 mJ) patients (low vs. intermediate: log-rank test, χ^2^ = 5.020, *p* = 0.019; low vs. high: log-rank test, χ^2^ = 6.026 *p* = 0.014; Table 5, Fig. 4b).

**Table 4:** Univariate and multivariate Cox regression analyses for factors affecting pain outcome

	Univariate Analyses			Multivariate Analysis		
	HR	95% CI	*p*-value	HR	95% CI	*p*-value
Age	0.994	0.977–1.011	0.491			
Gender	1.195	0.725–1.970	0.484			
Volume	0.956	0.933–0.980	0.000			
Integral dose	0.549	0.381–0.792	0.001	0.540	0.376–0.775	0.001
D_mean_	1.027	0.987–1.068	0.189	1.041	1.002–1.080	0.038
Isodose	0.988	0.908–1.075	0.777			
D_max_	1.026	0.991–1.062	0.150			
Prescription dose	1.578	0.984–1.020	0.209			
Re-irradiation yes/no no as reference	0.732	0.315–1.699	0.467			
Multiple sclerosis yes/no no as reference	0.340	0.162–0.714	0.004	0.332	0.156–1.002	0.004

**Table 5:** Comparisons of binarized treatment parameters for the probability of maintaining pain relief

Variables	n	Chi^2^ and *P*-values
		Pain
Volume (*<* 30 vs. *≥* 30 mm^3^)	205 vs. 91	χ^2^ = 4.675, p = 0.031
Integral dose (< 1.4, 1.4–2.7, > 2.7 mJ)	114 vs. 144 vs. 39	low vs. intermediate: χ^2^ = 5.020, *p* = 0.019 low vs. high: χ^2^ = 6.026, *p* = 0.014 intermediate vs. high, χ^2^ = 0.221, *p* = 0.638
D_mean_ (< 63 vs. *≥* 63 Gy)	75 vs. 221	χ^2^ = 1.803, *p* = 0.179
D_max_ (< 72 vs. *≥* 72 Gy)	132 vs. 164	χ^2^ = 2.756, *p* = 0.097
Prescriptin dose (< 58 vs. *≥* 58 Gy)	130 vs. 166	χ^2^ = 0.440, *p* = 0.838

*p* < 0.05 - significant, log-rank test. χ^2^ - test statistic of the Chi^2^ test

#### Occurrence of sensory disturbances

Re-irradiation using CyberKnife-SRS and presence of MS were independent predictors for the development of bothersome post-treatment numbness in the univariate and multivariate analysis. We also detected a minor but still significant association of isodose in the univariate analysis (Table 6). As with the pain relief analysis, we chose to assess D_mean_ in the multivariate analysis and had to exclude D_max_ due to multicollinearity. When patients with re-irradiation and/or MS were excluded, the multivariate model showed slight adjustments in the HR estimators. Isodose became a significant predictor variable with a weak influence (Isodose: HR = 1.097, CI = 1.004–1.198, *p* = 0.041).

**Table 6:** Univariate and multivariate Cox regression analyses for factors affecting the occurrence of numbness

	Univariate Analyses			Multivariate Analysis		
	HR	95% CI	*p-*value	HR	95% CI	*p*-value
Age	0.994	0.978–1.012	0.519			
Gender	0.906	0.566–1.450	0.681			
Volume	0.987	0.970–1.005	0.148			
Integral dose	0.787	0.594–1.044	0.097	0.789	0.603–1.033	0.085
D_mean_	0.973	0.937–1.010	0.155	1.024	0.986–1.064	0.214
Isodose	1.106	1.016–1.204	0.021	1.072	0.978–1.176	0.136
D_max_	0.969	0.938–1.002	0.062			
Prescription dose	0.977	0.836–1.019	0.281			
Re-irradiation yes/no no as reference	4.446	2.667–7.412	0.000	5.814	3.252–10.295	0.000
Multiple sclerosis yes/no no as reference	0.390	0.167–0.908	0.024	0.196	0.079–0.484	0.000

## Discussion

### Key results

This study was aimed at identifying irradiation parameters that may predict efficacy and safety of image-guided non-isocentric radiosurgery for treatment of TN. For this purpose, we analyzed the impact of target nerve volume, target dose, and the dose/volume relationship, together with relevant clinical characteristics, in a large multicenter German-Italian cohort of patients with a follow-up interval of up to 10 years. As expected, smaller target volume and lower ID were associated with worse pain outcome. In particular, we suggest that a target volume around 30 mm^3^ and an ID > 1.4 mJ offer better outlooks of sustained pain control. MS as a cause of TN was an independent prognosticator of treatment failure and shorter pain free interval. Furthermore, re-irradiation and presence of MS were independent predictors of the occurrence of numbness.

The results of this series in terms of pain relief are consistent with those previously reported and suggest that image-guided robotic radiosurgery represents an effective treatment of TN [7, 8, 10, 17]. Our results are indeed slightly superior to those previously published by Romanelli et al. (*n* = 138 pts.), who reported 76% pain relief after 3 years compared to 81.4% in the present cohort [7]. This comparison remained the same with the more recent series by Romanelli et al., with 76% pain relief at 36 months [27]. Furthermore, we here report results with longer follow-up and describe actuarial pain control rates (BNI class I-III) at 6, 12, 24, 36, 48, and 60 months of 96.8, 90.9, 84.2, 81.4, 74.2, and 71.2%, respectively (Fig. 2). With regard to sensory disturbances, facial numbness was reported by 18% of patients, with < 1% reporting bothersome hypoesthesia. Our results are also similar to those published by the two largest studies on GKS [10, 17]. In particular, Regis et al. have recently published the long-term results of 497 patients with the probability pain relief at 6 months and 1, 2, 3, 5, 7, and 10 years of 94.3, 90.2, 87.7, 83.6, 80.3, 75.4, and 67.7%, respectively, and a rate of new sensory disturbances of 20.4% [28].

### Interpretation

Tuleasca et al. summarized various treatment regimens regarding dose and target delineation of SRS in the treatment of TN [8]. Romanelli et al. was the first to report the use of frameless and non-isocentric SRS as a treatment option for TN [29]. They reported high precision and almost immediate pain relief following treatment with a PD between 65 and 70 Gy on a nerve segment up to 11 mm [29]. However, the irradiation of such long nerve segments led to a significant numbness in these patients; thus, new therapeutic algorithms were explored to identify the best treatment parameters.

In 2008 Villavicencio et al. [22] published the evaluation of 95 patients who were treated using CyberKnife-SRS. The authors included not only heterogeneous treatments in this study concerning dose and target volumes, but also modalities like isocentric and non-isocentric. The median maximal dose used was 75 Gy. Some variables were predictive for pain recurrence, such as median maximum and minimum dose and targeted median nerve length. In this cohort, 50% of the population had good pain control after 2 years, but 47% of the patients developed new facial numbness [22]. Thus, the irradiation of a long trigeminal nerve segment led to a very good rate of pain control, but also to a significant risk of facial numbness and suggested the necessity to set precise dose/volume constraints to optimize treatments.

Romanelli et al. have recently found a shorter treated nerve length (< 6 vs. 6 mm), a smaller treated nerve volume (< 30 vs. > 30 mm^3^), and a lower PD (< 58 vs. > 58 Gy) as predictors for treatment failure in univariate analysis, while none of these factors remained significant in multivariate analysis [7]. Those results were integrated into a larger and multicentric population of patients to improve the reliability and reproducibility of the findings. In the present univariate analysis, target nerve volume, ID, and presence of MS turned out to be factors associated with pain recurrence. ID and MS also retained significance in the multivariate analysis, while volume could not be included because of high correlation with ID. Our results support the impact of MS on the outcome of patients with TN, consistent with our previous study where only 44% of patients were pain-free for years after therapy [30]. This present study confirmed that patients with nerve volumes < 30 mm^3^ were more likely to suffer pain relapse. In addition, this was also observed for patients with low ID (< 1.4 mJ). The ID is the product of the mean dose and the target nerve volume and therefore represents an ideal parameter for non-isocentric treatments in which not only the dose, but also the target volume can be modulated to fit individual anatomy and clinical objectives. Mousavi et al. [23] investigated 155 patients treated by GKS and reported that the optimal treatment outcome could be achieved by medium IDs (1.4–2.7 mJ). To be able to compare our cohort with the study by Mousavi et al. [23], we categorized the ID in the same way and observed that the patients with lower IDs (i.e. < 1.4 mJ) were more likely to suffer pain relapse. This is in line with our finding that lower ID was an independent predictor for pain recurrence in this analysis. One treatment parameter which has been associated with the clinical outcome, particularly to the risk of sensory disturbance, is the dose received by the brainstem [21, 31].

Finally, we identified re-irradiation as the major independent predictor for sensory disturbance, with almost a six times higher risk after CKS as already described by Romanelli et al. [7] In our univariate analyses, presence of MS and prescription isodose, in addition to re-irradiation, were also associated with the development of sensory disturbances, but only re-irradiation and MS retained significance in the multivariate analysis as independent prognostic factors.

### Limitations

Our follow-up length is longer than that of most studies focusing on the efficacy of SRS in TN published so far [9–17], but much longer follow-up periods are needed for a definitive evaluation of the technique. This is indeed essential to provide a valid comparison with MVD. Although we have reported good to excellent outcomes, long-term results up to 10 years later could be disappointing, with up to 60% pain recurrence rates at a later stage [17, 28, 32].

## Conclusions

This study represents the largest multicenter frameless CKS series conducted to analyze possible treatment parameters as predictors of outcome in addition to the description of efficacy and safety profile of the technique. Our study confirmed that single treatment parameters are not enough to predict treatment efficacy of SRS in TN. The ID, D_mean_, and nerve volume are apparently relevant, as they may differ significantly in treatments using apparently similar dose and volume constraints. Our study supports the concept of personalized radiosurgery for the best treatment outcome and warrants future prospective studies.

## Supplementary information

**Additional file 1.**

## Data Availability

The datasets used and/or analyzed during the current study are available from the corresponding author on reasonable request.

## Abbreviations

SRS: Stereotactic radiosurgery
TN: Trigeminal neuralgia
MS: Multiple sclerosis
BNI: Barrow Neurological Institute
MVD: Microvascular decompression
GKS: Gamma knife radiosurgery
CKS: CyberKnife system
D_mean_: Mean treatment dose
D_max_: Maximal treatment dose
PD: Prescription dose
ID: Integral dose
CT: Computed tomography
CISS: Co-registered constructive interference in steady state
MR: Magnetic resonance
OAR: Organs at risk
HR: Hazard ratio
CI: Confidence interval
