# Supplementary material for: Factors affecting outcome in frameless non-isocentric stereotactic radiosurgery for trigeminal neuralgia: a multicentric cohort study
Source: Radiat Oncol. 2020 May 22;15:115. doi: 10.1186/s13014-020-01535-1 (PMC7243318; doi:10.1186/s13014-020-01535-1)
Supplement: Supplementary file 1 — Additional file 1. [file 13014_2020_1535_MOESM1_ESM.docx]

**Additional file 1**

**Radiosurgery treatment**

Patients were treated by SRS using a CyberKnife device (Accuray Inc., Sunnyvale, CA). Before treatment, a thermoplastic mask for immobilization during irradiation was individually created for each patient. Then, high resolution thin-sliced images (0.75–1.0 mm) were generated using computed tomography (CT) with this mask on. The treatment was planned on a native CT scan and co-registered constructive interference in steady state magnetic resonance (MR) images using MultiPlan (Accuray Inc., Sunnyvale, CA; figure 1). The planning of the treatment was as described previously.[^1-5^](#_ENREF_1)

**Selection of the target and dose**

Target and dose selection were performed as described in our previous studies.[^1-5^](#_ENREF_1) The Gasserian ganglion and the retrogasserian portion of the trigeminal nerve and Meckel’s cave were identified on the MR and CT images. A nerve segment of 5 to 6 mm, along with its lateral margins, was contoured on 2 to 5 slices depending on the anatomy of the nerve.

Critical structures such as the brainstem, the mesial temporal lobe, the acoustic and facial nerves, the cochlea, and the semicircular canals were marked to minimize radiation dose.

The dose distribution was calculated using the Ray-Tracing algorithm and optimized using an inverse sequential algorithm. In routine treatment planning, beams through the eyes were not allowed. The smallest available collimator and the shortest source-to-axis distance were selected, providing an effective collimation diameter of 4 mm at the alignment center.

Once the calculation was obtained, we checked that a 4 to 6 mm nerve segment was within the prescription isodose line (figure 1). Dose constraints for the brainstem for single fraction CyberKnife radiosurgery were as follows: equal to or less than 0.35 cm³ of the medulla could receive 10 Gy; $\leq$1.2 cm³ of the medulla could receive a threshold dose of 7 Gy with a maximum point dose of 14 Gy in 0.035 cm³ or less. Doses to the internal auditory canal, cochlea, labyrinth, and mesial temporal lobe were accurately reviewed, and the plan was changed accordingly if critical doses were found to overlap these structures. Overall the treatment procedure lasted 45–90 mins.

**Patient preparation and treatment session**

Patients were positioned on the treatment couch using individual masks, which were created during the CT procedure. After an initial alignment of the patient with in-room lasers that define the center of the system, the patient’s skull was tracked using x-ray images. The target location system (TLS) compares orthogonal x-ray images, called “live images”, obtained during patient set up with digitally reconstructed radiographs obtained from the planning CT scan. X-ray energy, mA, and exposure time were adjusted to optimize the quality of the live images. From the image comparison, the system calculates the deviation between the current patient position and the patient's position during the CT and suggests correction shifts and rotations, which are confirmed and initiated by the therapists. The patient was then iteratively aligned to the final treatment location by a robotic couch. When the residual displacements were within acceptable values of <1 mm and <0.5°, the irradiation was started, where remaining translational errors were corrected by the robot. The TLS enables x-ray imaging and confirmation of the patient position at defined time intervals. For the treatment of trigeminal neuralgia, a frequency of 15 to 60 s was used to reduce the intrafractional inaccuracy. Any detected deviation between the patient’s position during CT and that at the time of treatment was corrected by robot-assisted position adjustments.

**Quality assurance**

The targeting accuracy of the 6D Skull Tracking mode was verified monthly irradiating Gafchromic EBT3 films placed in an anthropomorphic phantom of the head (Ashland Advanced Materials, Bridgewater, NJ). The measured total target error of these end-to-end tests was always ≤0.95 mm. Quality of MR images was also verified monthly.

**References**

1 Conti A, Pontoriero A, Iati G, Esposito F, Siniscalchi EN, Crimi S, et al. Frameless stereotactic radiosurgery for treatment of multiple sclerosis-related trigeminal neuralgia. World Neurosurg. 2017;103:702-12.

2 Conti A, Pontoriero A, Midili F, Iati G, Siragusa C, Tomasello C, et al. CyberKnife multisession stereotactic radiosurgery and hypofractionated stereotactic radiotherapy for perioptic meningiomas: intermediate-term results and radiobiological considerations. Springerplus. 2015;4:37; doi:10.1186/s40064-015-0804-2.

3 Conti A, Pontoriero A, Ricciardi GK, Granata F, Vinci S, Angileri FF, et al. Integration of functional neuroimaging in CyberKnife radiosurgery: feasibility and dosimetric results. Neurosurg Focus. 2013;34:E5; doi:10.3171/2013.2.FOCUS12414.

4 Conti A, Pontoriero A, Salamone I, Siragusa C, Midili F, La Torre D, et al. Protecting venous structures during radiosurgery for parasagittal meningiomas. Neurosurg Focus. 2009;27:E11; doi:10.3171/2009.8.FOCUS09-157.

5 Conti A, Pontoriero A, Siddi F, Iati G, Cardali S, Angileri FF, et al. Post-treatment edema after meningioma radiosurgery is a predictable complication. Cureus. 2016;8:e605; doi:10.7759/cureus.605.
